# Unusual Hand Radiographic Presentation in a Patient on Hemodialysis

**DOI:** 10.4084/MJHID.2012.0009

**Published:** 2012-01-25

**Authors:** Chrysoula Pipili, Eirini Grapsa, Helen Tzanatos

**Affiliations:** Department of Nephrology, Aretaieion University Hospital, Athens, Greece

**Dear Editor**,

We read with great interest the report of Alcelik et al.^1^ (May 16 issue 3: e2011022, DOI 10.4084/MJHID.2011.022), about the case of upper extremity thrombosis caused by hand knitting. Knitting is also a popular Greek leisure activity, which gratify women of all ages, even when they suffer from serious health problems. Repetitive minor injuries caused from knitting may result in uncommon and challenging clinical features, requiring special consideration. We report the unusual bone alterations on a woman receiving hemodialysis, presenting a history of prolonged knitting.

## Case

**Figure 1:**
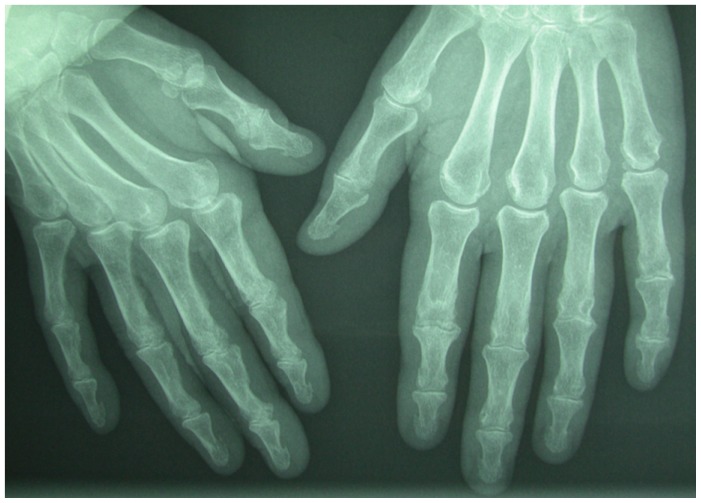
Hand radiograph enhanced by using mammography film (showing subperiostal bone resorption at the inner part of the terminal thumb phalanges symmetrically)

This 64-year old woman with chronic kidney disease due to nephrosclerosis, commencing on hemodialysis before 3 years, desired to candidate for kidney transplantation. Her medical history limited to hypertension of 10 years duration and end stage renal disease with well controlled secondary hyperparathyroidism [intact parathyroid hormone (iPTH): 17O pg/ml], but increased calcium phosphorus product (about 62 over the last 6 months) due to non compliance with absorption of phosphorus binders. No signs of osteoporosis have been identified on Dexa scan. Pretransplantation evaluation of the hands (plain radiograph) identified subperiostal bone resorption at the inner part of the terminal thumb phalanges symmetrically at both hands, with preservation of peripheral cortex (figure 1). Moreover, parathyroid ultrasound and radionuclide scanning with Tc-99m-sestamibi as well, revealed hyperplasia of bottom parathyroid glands. Although secondary hyperparathyroidism had been documented, the radiographic findings of the hands were not typical. Indeed, further enquiry disclosed knitting as hobby activity for 20 years disrupted with the start of dialysis.

## Discussion

Published data point out that the earliest subperiostal bone resorption due to secondary hyperparathyroidism is demonstrated at the phalangeal tufts and the radial aspects of the middle phalanges of the second and the third hand fingers. Whereas previously, radiologic findings were more intense, improvements in therapeutic management of secondary hyperparathyroidism in hemodialysis result in significant decrease of radiologic evidence.^2^,^3^ In our case, bone alterations on hands are not typical of renal osteodystrophy. The repeated and the prolonged abrasion between both thumbs during knitting are more likely compatible with traumatic bone defect (similar case has not been reported on bibliography). However, the role of dialysis osteodystrophy cannot be underestimated, probably acting as an additional factor to these lesions.
